# Morphological change of the posterior pole following the horizontal strabismus surgery with swept source optical coherence tomography

**DOI:** 10.1038/s41598-021-03351-3

**Published:** 2021-12-14

**Authors:** Yooyeon Park, Yong Chan Kim, Ye Jin Ahn, Shin Hae Park, Sun Young Shin

**Affiliations:** 1 Department of Ophthalmology, Dankook University Hospital, Cheonan, Republic of Korea; 2 Department of Ophthalmology, Incheon St. Mary’s Hospital, College of Medicine, The Catholic University of Korea, Seoul, Republic of Korea; 3 Department of Ophthalmology, Seoul St. Mary’s Hospital, College of Medicine, The Catholic University of Korea, Seoul, Republic of Korea

**Keywords:** Muscle, Experimental models of disease

## Abstract

Extraocular muscle movement during strabismus surgery causes changes in eyeball shape. Because extraocular muscle insertion is in front of the equator, it is thought that changes due to strabismus surgery mainly occur in the anterior segment. However, changes in the posterior segment of eye may also occur, which may also result in changes in refractive error after strabismus surgery. Using a 3-dimensional reconstruction technique (en face imaging) of the swept source optical coherence tomography, we determined and quantitatively measured the posterior polar change. The deepest interface between Bruch’s membrane and the choroid could be identified as the deepest point of the eyeball (DPE), and the location of the DPE relative to the optic disc and the fovea was measured. After lateral rectus muscle recession, the DPE moved away from the fovea, but after medial rectus muscle recession, the DPE moved toward the fovea. The amount of DPE movement differed by age and preoperative refractive error. Our findings suggest that the positional shift of the rectus muscle in horizontal strabismus surgery causes a structural change in the posterior segment of the eye, and the postoperative refractive changes may be related to this shift.

## Introduction

**Figure 1:**
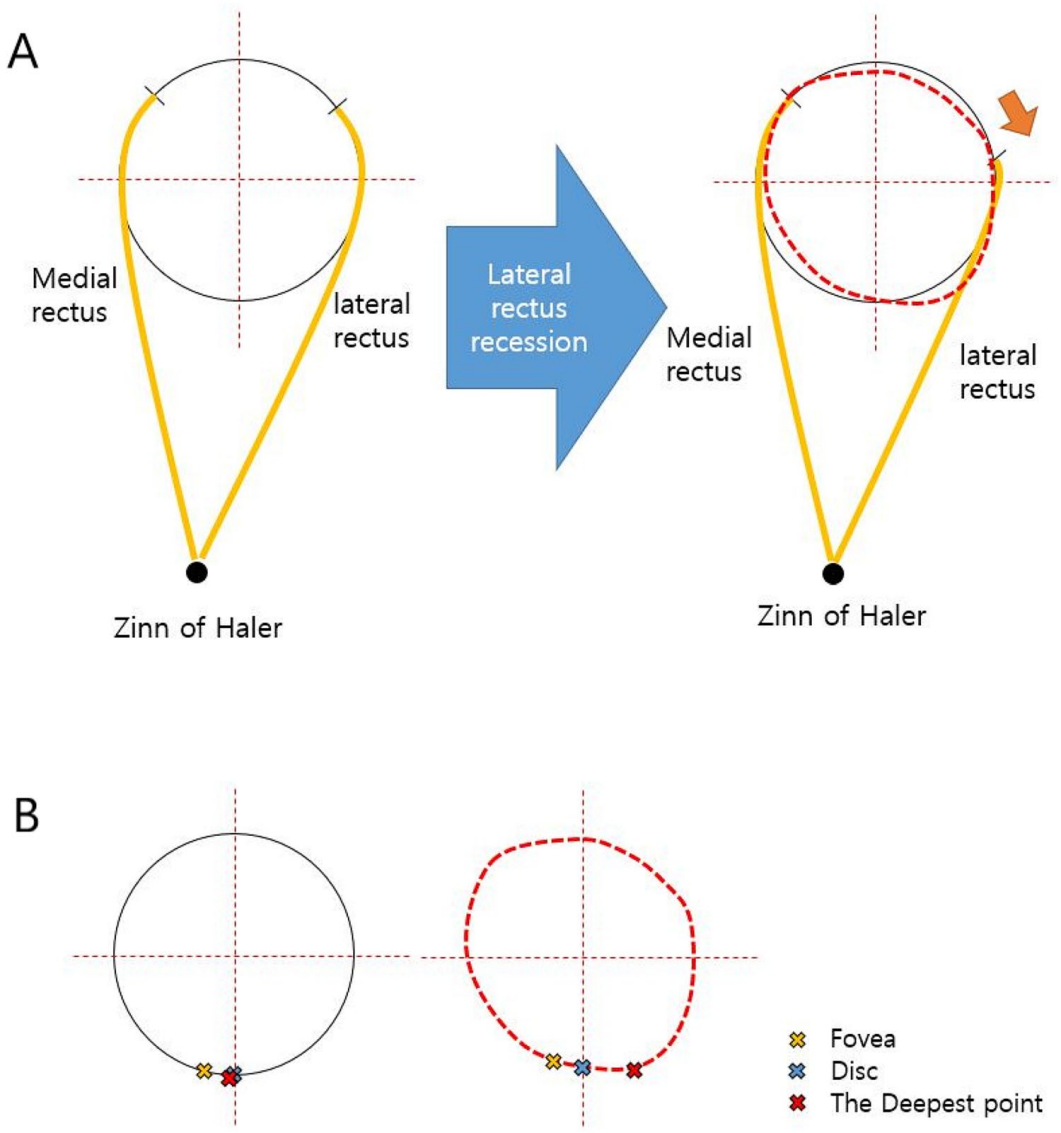
Schematic diagrams illustrating the shape of the eyeball alters after the strabismus surgery. (**A**) Shows the altered eyeball shape by dotted line with red color. (**B**) shows the schematic representation of the change in three parameters.

Changes in refractive error after strabismus surgery have been reported in the literature^1–6^. The amount of refractive error change and whether the change is transient are still debatable. Extraocular muscle force was thought to be an important contributing mechanism for refractive error change in previous reports^4,7–9^. Altering the locations of extraocular muscle insertions with strabismus surgery could change the tension or power vector of extraocular muscles and the shape of the eyeball^10^. It results in the morphological changes in not only the anterior part of the eyeball but also the posterior part (Fig. 1). However, to our knowledge, only anterior segment changes (e.g. anterior chamber depth or volume, corneal topographic measurements) have been reported^4,8,9^.

Recently, swept source optical coherence tomography (SS-OCT) has been developed and used for deep imaging of the posterior pole to choroid and even sclera. Named ‘en face’ imaging, using deep penetrance of the swept-source light to the choroid and scleral layer in combination with B-scan, it is possible to obtain a three-dimensional (3D) visualization of the posterior pole^11^. Through en face imaging, Kim et al.^12^ first introduced the concept of ‘deepest point of the eyeball (DPE)’. They found that by analyzing consecutive images of the coronal section around the posterior pole, the deepest interface between Bruch’s membrane and the choroid were identified as a decreasing plane, indicating DPE.

Considering the ocular shape of high myopic eyes with posterior staphyloma, which may be the ectasia expanded around the area of the DPE, the factors such as increased axial length, advanced patient’s age^13^, asymmetrical thinning of scleral structure presented mostly in inferior part where the embryonic ocular fissure closes^14^ may influence the position of DPE. The extraocular muscles, inserted on the sclera through their pulleys, can distort and displace the globes^15^. Therefore, we hypothesized that horizontal rectus muscle surgery may influence on the DPE location and the shape of the posterior part of eyeball.

The purpose of this study was to investigate 3D morphological changes in the posterior part of the eyeball after horizontal rectus muscle recession by analyzing alterations in the relative positional relationships of the DPE, fovea, and optic disc using the SS-OCT en face analysis.

## Results

A total of 110 eyes from 55 enrolled patients were analyzed. Eyes were classified into three groups according to the surgical management: a lateral rectus muscle (LR) recession group that had preoperative exotropia (71 eyes); a medial rectus muscle (MR) recession group with preoperative esotropia (19 eyes); and a control group of unoperated fellow eyes, which were the other eyes when unilateral surgery was performed (20 eyes). Unilateral LR or MR recession was performed on the chief-deviated eye (CDE).

Table 1 shows the basic characteristics of the enrolled patients. The mean patient age was 21.2 ± 18.1 years old (range 7 to 65 years old). In the LR recession group, preoperative exotropia amount was average 28.0 prism diopters (PD). The average recession amount was 7.13 ± 2.27 mm. After the surgery, patients showed an average 0.9 ± 5.4 PD of esophoria on the day of operation, which was considered a successful overcorrection amount. The MR recession group showed similar amounts of preoperative esotropia with 27.4 ± 13.0 PD, which was corrected to an average 0.6 ± 2.7 PD of exophoria after 6.19 ± 0.69 mm of MR recession. There was no significant differences in gender distribution and CDE.

**Table 1:** Baseline characteristics of enrolled patients.

	LR recession (N = 41, n = 71)	MR recession (N = 14, n = 19)	Control (N = 20, n = 20)	p-value^†^
**Age (year)**	18.2 ± 15.3	28.3 ± 24.6	24.85 ± 18.30	0.4
Adult (≥ 20 year)	37.5 ± 15.6	51.6 ± 21.7	36.5 ± 17.4	0.499
Child (< 20 year)	10.1 ± 2.7	11.3 ± 2.7	10.7 ± 1.9	0.527
**Gender, n, (%)**				
Male	23 (56.1%)	5 (35.7%)	9 (45.0%)	0.469
Female	18 (43.9%)	9 (64.3%)	11 (55.0%)	
SE (D)	− 1.89 ± 3.10	+ 0.24 ± 2.57	− 1.04 ± 4.15	0.107
**Angle of deviation (PD)**				
Preop	28.0 ± 11.2	27.4 ± 13.0		0.905
Postop	− 0.9 ± 5.4*	− 0.6 ± 2.7*		0.487
**CDE, n (%)**				
Right eye	24 (58.5%)	8 (57.1%)		0.684
Left eye	17 (41.5%)	6 (42.9%)		
Recession amount of muscle (mm)	7.13 ± 2.27	6.19 ± 0.69		0.297
Corrective effect of muscle (°/mm)	2.47 ± 0.73	2.92 ± 0.62		0.022

Mean ± SD, otherwise indicated.

*LR* lateral rectus muscle, *MR* medial rectus muscle, *SE* spherical equivalent, *D* diopter, *PD* prismatic diopter, *CDE* chief deviated eye.

*Postop angle of deviation was presented as minus values when overcorrection was observed by alternative prism cover test.

^†^Kruskal–Wallis test for longitudinal data, chai-square test for categorical data.

**Figure 2:**
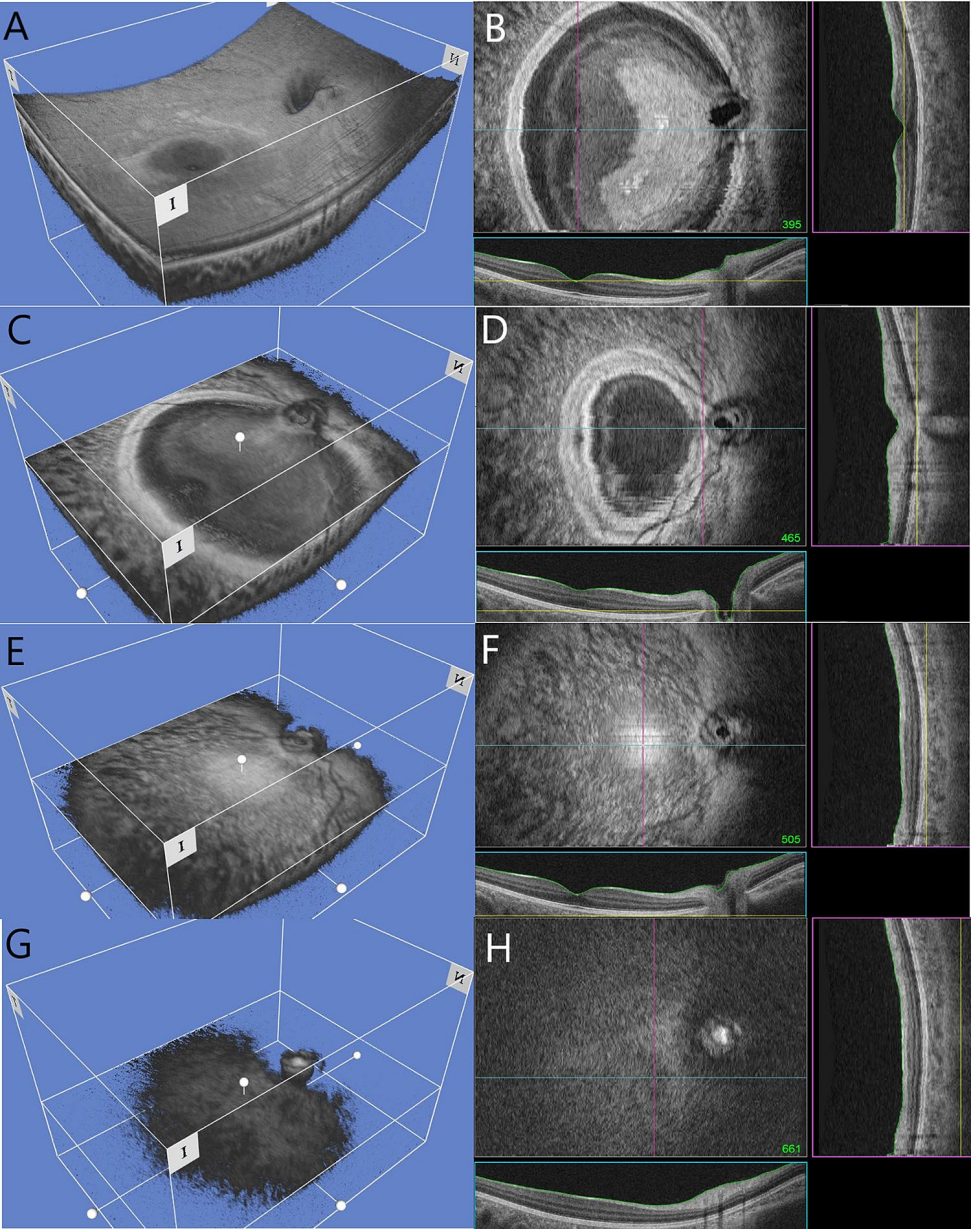
Representative three dimensional (3D) reconstructed images by swept source optical coherence tomography (en face image). (**A**,**C**,**E**,**G**) represents 3D reconstructed images and (**B**,**D**,**F**,**H**) represents the 2D B-scan image with vertical and horizontal cross sections. (**A**) and (**B**) shows fovea level. (**C**) and (**D**) shows disc center level, bruch’s membrane level of nasal border of the optic disc. (**E**) and (**F**) shows retinal DPE level, which is the border of outer retina and bruch’s membrane. (**G**) and (**H**) shows choroid DPE level, where the border of choroid and inner sclera meets.

**Figure 3:**
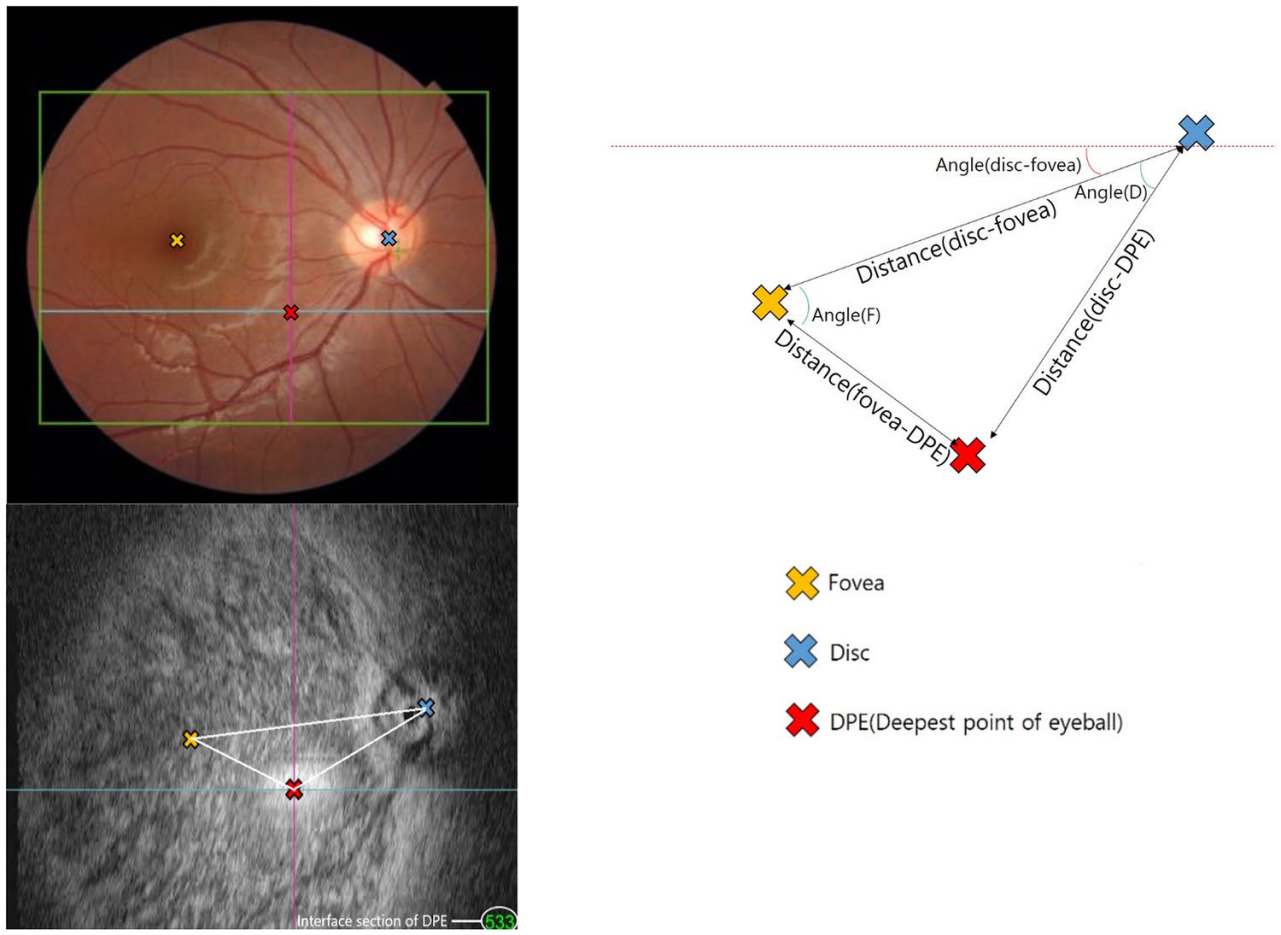
Schematic diagram of the location of disc center, fovea and the DPE. Fundus photograph (left upper) and B-scan of the same patient (left lower) shows the real location of three parameters. The depth of the B-scan image can be calculated with a section number showed in right lower corner on B-scan image.

Disc and foveal location were confirmed as base points in order to measure changes in the location of DPE. The DPE was defined as the retinal DPE (rDPE) where Bruch’s membrane and the choroid interface become one spot and as the choroidal DPE (cDPE) where the choroid and scleral anterior margin become one spot (Figs. 2, 3). There was no significant differences in the 3D parameters (distance, angle, depth) for disc to foveal location (Table 2).

**Table 2:** Preoperative and postoperative values of Disc and foveal location of lateral rectus recession group, medial rectus recession group, and control.

Disc-Fovea	LR recession	MR recession	Control	p-value*
**Distance (μm)**				
Preop	4929.36 ± 362.95	4984.24 ± 368.78	5020.75 ± 506.38	0.680
Postop	4922.74 ± 371.50	5008.41 ± 286.57	4970.13 ± 430.07	0.684
p-value^†^	0.906	0.586	0.569	
**Angle (°)**				
Preop	7.51 ± 5.10	10.59 ± 9.61	7.25 ± 4.88	0.132
Postop	8.13 ± 5.10	11.39 ± 8.61	6.48 ± 3.89	0.059
p-value^†^	0.212	0.08	0.313	
**Depth (μm)**				
Preop	53.12 ± 331.96	198.69 ± 293.26	138.06 ± 271.51	0.343
Postop	70.74 ± 284.24	219.63 ± 276.34	147.97 ± 245.00	0.099
p-value^†^	0.3	0.587	0.611	

Mean ± SD, otherwise indicated.

*LR* lateral rectus muscle, *MR* medial rectus muscle, *preop* preoperative value, *postop* postoperative value.

*One way ANOVA test for Distance, Kruskal–Wallis test for angle and depth.

^†^Paired t-test for Distance, Wilcoxon’s signed rank test for angle and depth.

**Figure 4:**
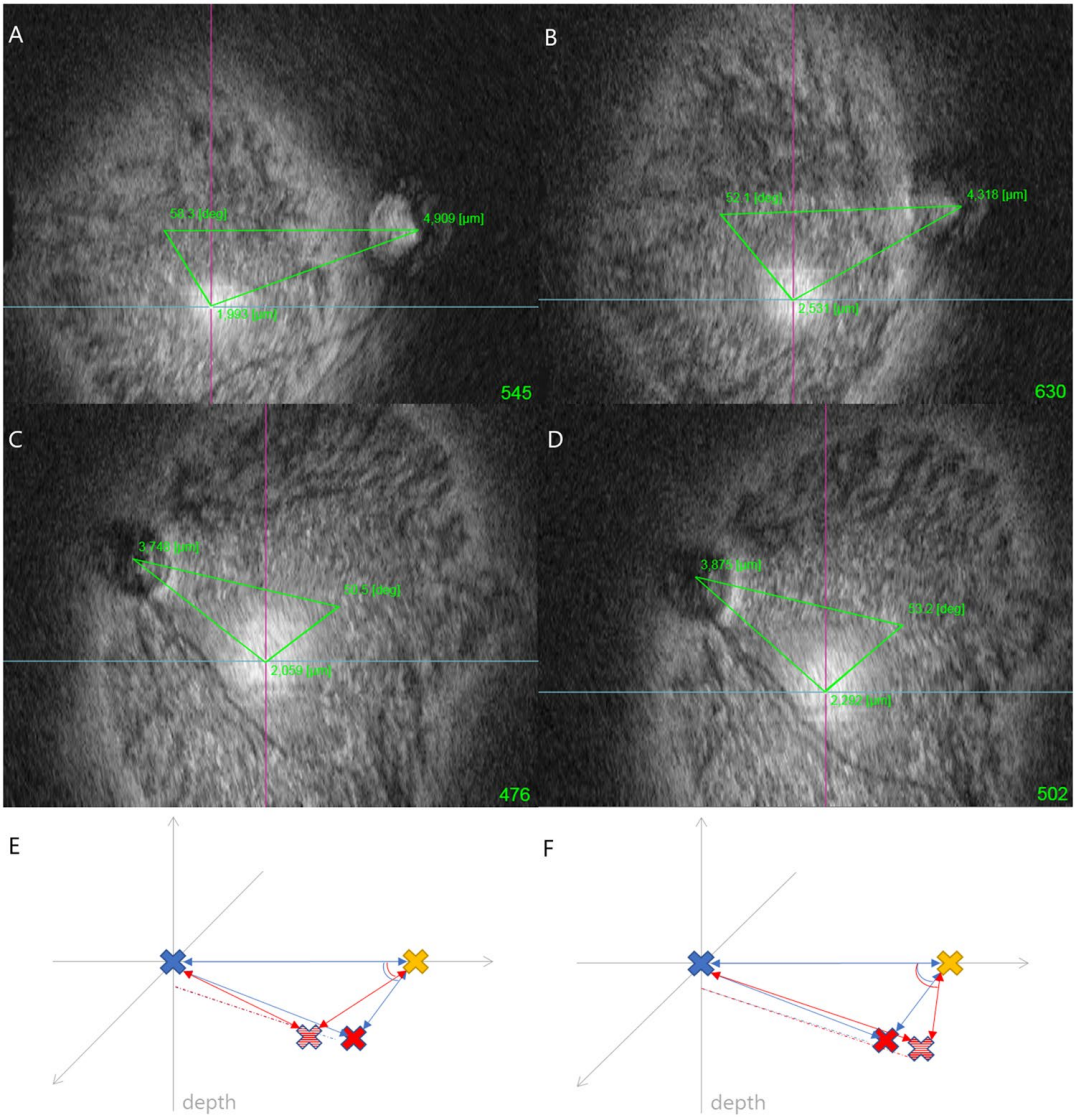
Representative data and schematic diagram of DPE change in exotropia (LR recession) and esotropia (MR recession) groups. Yellow colored X, fovea. Blue colored X, optic disc. Red colored X, DPE before operation. Stratified red colored X, DPE after operation. (**A**) Preoperative data of a patient in LR recession group. (**B**) Postoperative data in LR recession group. (**C**) Preoperative data and (**D**) Postoperative data in MR recession group. (**E**) Schematic diagram of LR recession group. After LR recession, DPE-fovea distance elongated and DPE-fovea angle become smaller. (**F**) Schematic diagram of MR recession group. After MR recession, DPE-disc distance elongated and DPE-fovea angle become larger.

In the control group, there were no significant differences before and after surgery in refractive errors and angle of the astigmatism axis or the rDPE and cDPE locations (Table 3). However, in eyes undergoing surgery, DPE location changed after surgical management. Table 4 shows the differences between preoperative and postoperative values of refractive error and DPE location. Some myopic shift after LR recession and hyperopic shift after MR recession was observed; the changes significantly differed between the groups (p = 0.009). rDPE showed no changes in the parameters, but the changes in cDPE-fovea distance and cDPE angle significantly differed between the groups (p = 0.022, 0.031, respectively). After LR recession, the cDPE moved away from the fovea and decreased in angle, but after MR recession, the cDPE moved towards the fovea and increased in angle (Fig. 4).

**Table 3:** Values of refractive errors and DPE locations before and after the surgery in control group.

Factors	Preop	Postop	p-value*
SE (D)	− 1.04 ± 4.15	− 1.09 ± 3.94	0.707
Cylinder (D)	− 1.08 ± 0.79	− 1.14 ± 0.82	0.460
Axis of cylinder (°)	127.45 ± 61.21	121.45 ± 65.93	0.519
**rDPE**			
Distance (μm)			
DPE-disc	3677.95 ± 1582.22	4192.90 ± 1160.49	0.056
DPE-fovea	2750.10 ± 1739.14	2455.35 ± 1501.74	0.1
Angle (°)			
DPE-disc	31.3 ± 25.9	26.6 ± 22.0	0.133
DPE-fovea	43.2 ± 37.2	55.9 ± 37.8	0.135
Depth (μm)			
DPE-disc	171.6 ± 159.1	176.5 ± 127.5	0.854
DPE-fovea	309.7 ± 195.5	254.5 ± 182.2	0.068
**cDPE**			
Distance (μm)			
DPE-disc	4446.95 ± 1172.24	4876.70 ± 894.91	0.064
DPE-fovea	2000.60 ± 1254.50	1979.30 ± 1246.51	0.824
Angle (°)			
DPE-disc	18.46 ± 13.42	21.87 ± 15.90	0.187
DPE-fovea	64.43 ± 32.88	79.89 ± 35.87	0.062
Depth (μm)			
DPE-disc	406.1 ± 185.4	455.5 ± 130.2	0.102
DPE-fovea	531.2 ± 195.0	503.5 ± 186.5	0.088

Mean ± SD, otherwise indicated.

*SE* spherical equivalent, *DPE* deepest point of the eyeball, *rDPE* retinal DPE, *cDPE* choroidal DPE.

*Wilcoxon’s signed-rank test.

**Table 4:** Preoperative to postoperative value difference of refractive error and DPE parameters in LR recession and MR recession group.

Postop-preop difference	LR recession	MR recession	p-value^†^
SE (D)	− 0.42 ± 0.73	0.13 ± 0.37	0.009*
Cylinder (D)	− 0.33 ± 0.50	− 0.18 ± 0.42	0.259
Axis of cylinder (°)	12.62 ± 61.89	6.68 ± 70.76	0.720
**rDPE**			
Distance (μm)			
DPE-disc	− 45.35 ± 598.47	− 45.00 ± 1962.03	0.278
DPE-fovea	133.82 ± 623.90	107.15 ± 1051.04	0.42
Angle (°)			
DPE-disc	2.5 ± 1.8	8.2 ± 26.8	0.454
DPE-fovea	− 4.0 ± 18.9	− 13.4 ± 42.3	0.162
Depth (μm)			
DPE-disc	− 6.0 ± 81.0	26.0 ± 141.6	0.282
DPE-fovea	− 0.5 ± 77.4	26.4 ± 284.7	0.537
**cDPE**			
Distance (μm)			
DPE-disc	− 118.47 ± 769.24	97.00 ± 914.32	0.996
DPE-fovea	203.97 ± 922.16	− 570.69 ± 691.86	0.022*
Angle (°)			
DPE-disc	− 1.9 ± 17.9	− 2.2 ± 13.9	0.629
DPE-fovea	− 20.1 ± 42.5	9.8 ± 24.1	0.031*
Depth (μm)			
DPE-disc	− 16.9 ± 102.2	11.6 ± 163.6	0.402
DPE-fovea	− 0.2 ± 87.2	49.3 ± 298.4	0.281

Mean ± SD, otherwise indicated.

*LR* lateral rectus, *MR* medial rectus, *SE* spherical equivalent, *DPE* deepest point of the eyeball, *rDPE* retinal DPE; cDPE, choroidal DPE.

*The value was statistically significant (p < 0.05). †Mann–Whitney test was used for statistical analysis.

When comparing the parameters between adults and children with cut off value of 20-years-of-age (Table 5), the cDPE-disc angle and the DPE-disc depth of retina and choroid significantly differed between adult and child groups in the operated eyes (p = 0.010 in rDPE and 0.007 in cDPE, respectively). Distance parameters showed similar changes in the two groups. The DPE-disc depth change was more prominent in the child group than in the adult group. In child group, preoperative rDPE-disc depth was 224.1 ± 235.1 μm and postoperative depth was 184.0 ± 185.3 μm; preoperative cDPE-disc depth was 438.4 ± 182.0 μm and postoperative depth was 393.3 ± 154.9 μm (p = 0.027 and p = 0.054, respectively, by Wilcoxon’s signed rank test).

**Table 5:** Comparison of preoperative to postoperative value difference between adult and child group separated by median age in operated eyes.

Postop-preop difference	Adult (N = 20)	Child (N = 35)	p-value^†^
SE (D)	− 0.46 ± 0.72	− 0.26 ± 0.68	0.208
Cylinder (D)	− 0.15 ± 0.47	− 0.37 ± 0.48	0.043*
Axis of cylinder (°)	− 1.83 ± 9.51	17.64 ± 72.99	0.054
**rDPE**			
Distance (μm)			
DPE-disc	42.81 ± 1009.43	− 14.68 ± 1179.52	0.137
DPE-fovea	132.16 ± 666.27	12.57 ± 820.59	0.833
Angle (°)			
DPE-disc	− 1.0 ± 17.2	3.0 ± 22.1	0.358
DPE-fovea	− 2.7 ± 32.3	− 4.5 ± 20.4	0.794
Depth (μm)			
DPE-disc	17.9 ± 111.2	− 40.1 ± 81.1	0.010*
DPE-fovea	− 6.8 ± 219.7	8.9 ± 110.5	0.156
**cDPE**			
Distance (μm)			
DPE-disc	72.88 ± 959.76	− 35.93 ± 740.08	0.569
DPE-fovea	− 48.44 ± 1222.08	34.89 ± 1131.70	0.791
Angle (°)			
DPE-disc	− 4.7 ± 21.5	4.7 ± 16.9	0.032*
DPE-fovea	− 11.4 ± 36.4	− 12.5 ± 38.1	0.807
Depth (μm)			
DPE-disc	42.7 ± 124.3	− 45.0 ± 109.1	0.007*
DPE-fovea	19.6 ± 239.5	− 13.6 ± 71.7	0.419

Mean ± SD, otherwise indicated.

*SE* spherical equivalent, *DPE* deepest point of the eyeball, *rDPE* retinal DPE, *cDPE* choroidal DPE.

*The value was statistically significant (p < 0.05). †Mann–Whitney test was used for statistical analysis.

**Figure 5:**
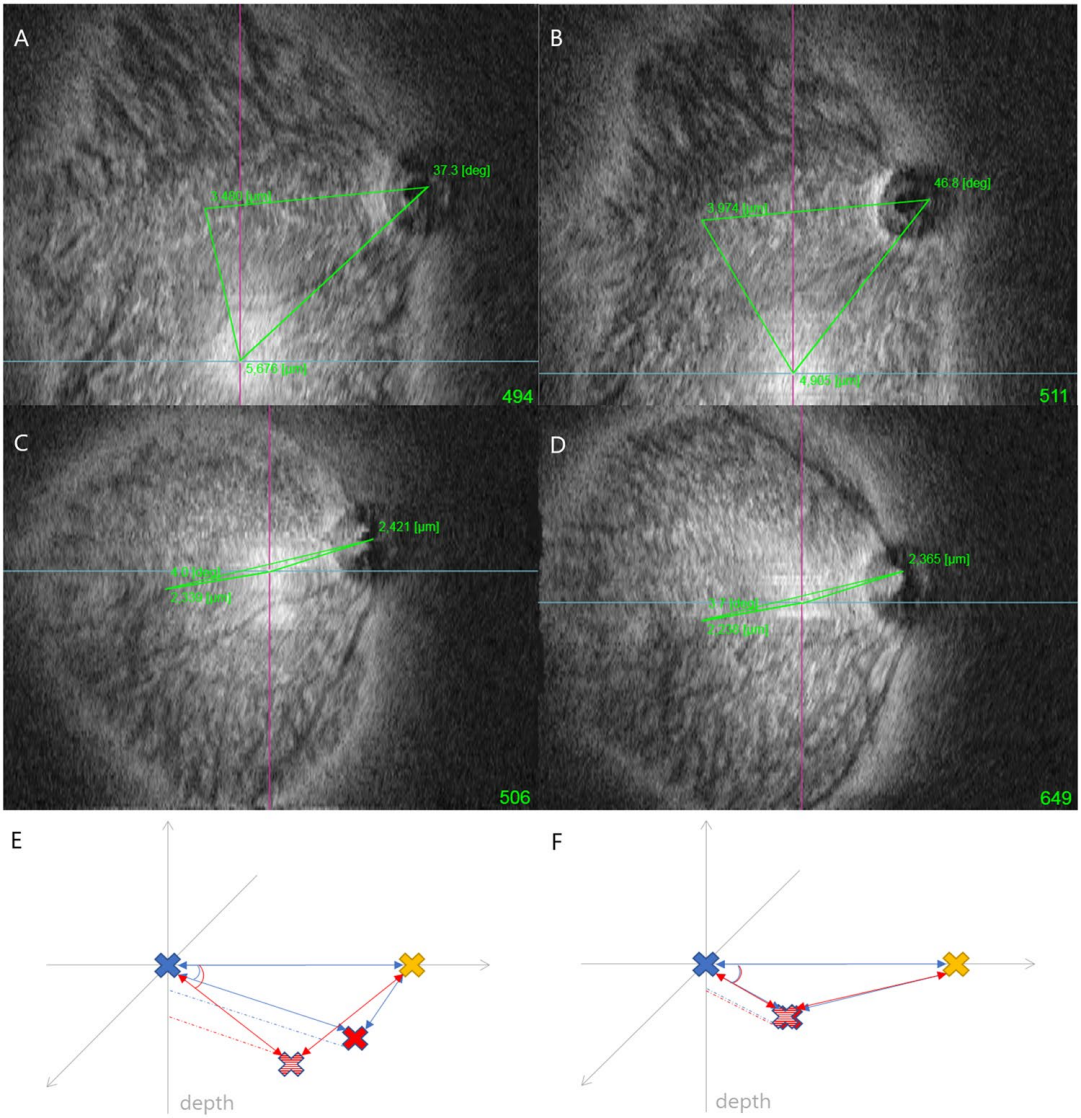
Representative data and schematic diagram of DPE change in myopia and non-myopia groups. Yellow colored X, fovea. Blue colored X, optic disc. Red colored X, DPE before operation. Stratified red colored X, DPE after operation. (**A**) Preoperative data of a patient in myopia group. (**B**) Postoperative data in myopia group. (**C**) Preoperative data and (**D**) Postoperative data in non-myopia group. (**E**) Schematic diagram of myopia group. In myopia group, DPE-fovea distance elongated, DPE-disc angle become larger. (**F**) Schematic diagram of non-myopia group. In non-myopia group, no statistical change noted.

Table 6 shows the differences in operated eyes between the myopia group with axial length (AL) over 24.0 mm and the non-myopia group with AL under 24.0 mm. Average preoperative DPE location significantly differed between the two groups. In the myopia group, DPE was near the fovea and distant from the disc. Conversely, DPE was located distant to the fovea and near the disc in the non-myopia group. Also, there was a significant postoperative change in the myopia group with the DPE moving further and deeper from the fovea (p = 0.017 and p = 0.037, respectively). However, there were no postoperative changes in DPE location in the non-myopia group. A schematic diagram of relative DPE locations in the two groups is shown in Figure 5.

**Table 6:** Comparison of values between preoperative myopia and non-myopia in operated eyes.

	Myopia (AL > 24.0 mm)	Non-myopia (AL ≤ 24.0 mm)	p-value^†^
AL (mm)	24.78 ± 0.85	20.36 ± 1.04	< 0.001*
**SE (D)**			
Preop	− 3.03 ± 2.11	1.47 ± 2.12	0.001*
Postop	− 3.26 ± 2.53	1.44 ± 2.24	
p-value	0.612	0.788	
**Cylinder (D)**			
Preop	− 1.44 ± 0.99	− 1.06 ± 1.24	0.082
Postop	− 1.62 ± 0.85	− 0.99 ± 0.91	
p-value	0.748	0.616	
**Axis of cylinder (°)**			
Preop	120.6 ± 75.2	122.5 ± 78.3	0.678
Postop	113.7 ± 78.8	117.4 ± 61.7	
p-value	0.22	0.433	
**Distance (μm)**			
DPE-disc			
Preop	3994.6 ± 1515.3	2805.0 ± 1689.2	0.073
Postop	3711.1 ± 1419.5	3073.5 ± 1555.7	
p-value	0.263	0.386	
DPE-fovea			
Preop	2371.8 ± 973.2	3823.4 ± 2299.8	0.05*
Postop	2735.9 ± 971.3	3619.3 ± 1950.9	
p-value	0.017*	0.445	
**Angle (°)**			
DPE-disc			
Preop	30.9 ± 11.7	37.0 ± 27.6	0.408
Postop	36.9 ± 15.4	44.7 ± 28.4	
p-value	0.093	0.386	
DPE-fovea			
Preop	45.6 ± 28.7	21.8 ± 28.5	0.065
Postop	39.4 ± 17.8	21.6 ± 14.9	
p-value	0.075	0.878	
**Depth (μm)**			
DPE-disc			
Preop	153.1 ± 131.0	69.6 ± 77.0	0.173
Postop	152.8 ± 154.6	51.6 ± 42.9	
p-value	0.889	0.447	
DPE-fovea			
Preop	297.7 ± 52.4	496.1 ± 232.3	0.021*
Postop	317.2 ± 45.5	412.9 ± 168.1	
p-value	0.037*	0.075	

Mean ± SD, otherwise indicated.

*AL* axial length, *SE* spherical equivalent, *DPE* deepest point of the eyeball, *D* diopters.

*The value was statistically significant (p < 0.05).

^†^Mann–Whitney test was used for statistical analysis. P value was analyzed for the postoperative–preoperative value change in each group.

## Discussion

This study revealed posterior pole changes in patients undergoing horizontal strabismus surgery by measuring the relative positions of the DPE, fovea, and optic disc with SS-OCT en face analysis. To our knowledge, this is the first study analyzing the effects of strabismus surgery on the posterior eye.

In our study, the positional relationship of the disc and fovea were consistent with previous reports. Normative cyclotorsion values have been reported as ranging from 6.1° to 7.3°^16,17^, and in a study of 50 patients with inferior or superior oblique overaction, Kushner^18^ had reported abnormal excyclotorsion as 13.96° ± 6.20° and incyclotorsion as 14.02° ± 7.03°. In our study, the average cyclotorsion amount was 7.25° in the control group, 7.51° in the LR recession group, and 10.59° in the MR recession group. Although the cyclotorsion values were relatively high in the MR recession group, the torsion values of all three groups were within physiologic range. Therefore we could use the fovea and disc as the reference points for determining relative DPE position.

After horizontal strabismus surgery, changes in extraocular muscle tension are thought to be an important mechanism underlying changes in refractive error and anatomical anterior segment parameters^8,9^. Previous studies focused on anterior segment changes after horizontal strabismus surgery^4,19,20^. Emre et al.^19^ described anterior chamber volume reduction one month after recession and resection in 18 eyes with either exotropia or esotropia. Jung and Choi^20^ found a one-month postoperative anterior chamber depth reduction in exotropia after lateral rectus recession surgery. Lastly, Noh et al.^4^ observed reductions in the center and temporal peripheral aspects of anterior chamber depth. These reductions were correlated with changes in corneal astigmatism toward the with-the-rule direction by flattening the horizontal meridian one week after LR recession, suggesting that the ciliary body may have altered the anterior chamber configuration.

In our study, changes in extraocular muscle tension seemed to also influence the posterior segment. After LR recession, the DPE location changed toward the disc far from the fovea with the depth between the fovea and the DPE increasing; however, we observed an opposite DPE location change in foveal direction after MR recession. Since the amount of strabismus correction was the same in both groups, the opposite direction change suggests a power vector influence of the extraocular muscle. In addition, the postoperative SE value changed to myopic in LR recession and hyperopic in MR recession, which suggests that 3D posterior pole changes affect the postoperative refractive error.

There were age-related differences in DPE-disc depth in the operated eyes. The adult group did not show significant changes but the child group showed decreases in DPE-disc depth. One possible explanation for the changes in depth is that when the power vector moves backward from the original insertion after horizontal muscle recession, the force would be more effectively transmitted to the posterior pole and pulled laterally to become thinner. In the child group, the depth change was more prominent in the retina-choroidal margin DPE (rDPE) than in the choroid-scleral margin DPE (cDPE). Disc-foveal depth did not change in either group.

Chen and Weiland had discovered that retinal elastin presented in human retinal vessels at the basement membranes and had found a correlation between decreasing elastin and moderate-to-severe age-related macular degeneration^21^. Liu et al.^22^ had also suggested that extracellular matrix remodeling of the optic nerve head by fibrotic tissue changes leads to age-related ocular stiffening and may be a potential cause of glaucoma. This suggests higher elasticity of the outer retina compared to the sclera in children.

In the present study, myopic eyes showed preoperative DPE locations closer to the fovea and farther from the disc than in non-myopic eyes, and the postoperative DPE changes were significantly more prominent in myopic eyes. This finding is consistent with previous studies from Yong Chan Kim et al.^12^ and Ohno-Matsui et al.^23^. In those previous studies, the eyeball elongated axially, the disc-DPE distance lengthened, the disc-DPE depth deepened, and the posterior pole showed a tendency to expand from the optic disc in the inferotemporal direction.

This study has some limitations. First, the values of SS-OCT en face images varied widely between individuals so statistical significance was difficult to obtain. However, we confirmed values in a blinded manner, and a previous study^12^ provided excellent reproducibility for DPE parameters, so the values are considered highly reliable. Second, a relatively small number of patients were enrolled due to difficulty in conducting SS-OCT in young patients and in other patients just after the surgery. Therefore, there is the sample size asymmetry. Maldonado et al.^24^ also pointed out the difficulty of OCT scanning with pediatric patients due to their inattention and movement. They used handheld probes to evaluate SD-OCT in neonates and emphasized that imagers should consider age and optics of the eye when setting image parameters in young patients. Third, the present study showed short-term morphological change of posterior pole within one week after the strabismus surgery. Some previous studies reported the recovery of anatomical changes of eyeball after one to several month postoperatively. Further prospective studies with long-term follow-up of postoperative period will be necessary to confirm if the parameters of posterior pole is critically associated with refractive change after the strabismus surgery. While it is easier to observe statistical significance between adults and young patients groups with larger sample sizes, even with our small sample size we could still achieve statistical significance in eyes undergoing strabismus surgery with posterior pole morphological changes and extraocular muscle power vector changes. These results provide another clue for posterior polar elasticity differences between age, and the axial elongation is consistent with previous studies.

In conclusion, en face imaging with SS-OCT enabled an approach to the posterior pole in the strabismus field. The positional shift of the rectus muscle after horizontal strabismus surgery is associated with a structural change in the posterior segment of the eye, suggesting that postoperative refractive changes may be related to this shift.

## Methods

### Study participants

This prospective cross-sectional study was conducted from Jun 2020 to December 2020 at the Seoul St. Mary’s Hospital of College of Medicine, The Catholic University of Korea, Seoul, Republic of Korea. Patients aged 6 to 70 years with horizontal strabismus and planned to receive LR recession for exotropia or MR recession for esotropia by a single surgeon (S.Y.S.) were enrolled in this study. Patients who were non-cooperative with the OCT examination were excluded. Patients with moderate to severe cataracts or corneal opacity, small pupils, or a visual acuity under 20/40 were also excluded due to low quality of obtained OCT images. Patients with ocular surgery history (including previous strabismus surgery) or retinal disease (including extremely high myopia with retinal degeneration) that could affect the posterior pole anatomy were excluded. Written informed consent was obtained from the participants, and also from their parents or guardians if their age is under 13 years old. The study was approved by the Institutional Review Board of Seoul St. Mary’s Hospital (IRB# KC20OISI0458), which followed the principles of the Declaration of Helsinki. This clinical study was registered in the public database of CRIS (The Clinical Research Information Service; https://cris.nih.go.kr).

Every surgery was performed by one of the authors (S.Y.S.). Each eye was assigned to either an LR recession group or MR recession group depending on the surgery procedure. The other eye (non-operated eyes) of operated eyes undergone unilateral LR or MR recession served as a paired comparison group to determine whether the posterior polar changes in the study eyes resulted from normal physiologic changes or were induced by surgery.

Each subject received a comprehensive ophthalmologic assessment, which included a medical history review, visual acuity measurement, slit-lamp biomicroscopy, an alternative prism cover test, non-cycloplegic refraction measurement with an autorefractor (RK-F10; Canon, Tochigiken, Japan), and AL determination using ocular biometry (IOL master; Carl Zeiss Meditec, Inc., Dublin, CA, USA) at the baseline visit. The posterior pole structure was obtained in three-dimensions (3D) by SS-OCT (DRIOCT Triton, Topcon Corporation, Tokyo, Japan). All subjects were then followed up with the same examination protocol at one week from the baseline visit to investigate the morphological changes of the posterior aspect of the eye under horizontal strabismus surgery.

### En face analysis of SS-OCT scans and measurement of the Deepest Point of the Eyeball (DPE)

A detailed analysis protocol has been previously described by Kim et al.^12^. With a long light beam wavelength of 1050 nm and a high penetration with 8 μm of axial resolution, we can more deeply visualize the choroid and sclera with SS-OCT than with spectral domain OCT (SD-OCT). En face images were obtained by 3D reconstruction using 1000 B-scans with 2.6 μm depth and 12 × 9 mm^2^ volumetric tissue scans. Topcon software (Topcon Corporation, Tokyo, Japan) was used for the 3D reconstruction.

Two dimensional (2D) or 3D coronal sections of each En face image can be seen simultaneously. Bruch’s membrane appeared as a hyper-reflective round plane, surrounded by the choroidal layer, which appeared as hypo-reflective scattered lesions. The intermediately-reflective scleral layer followed the choroidal layer (Fig. 2). The DPE was defined as the retinal DPE (rDPE) where Bruch’s membrane and the choroid interface became one spot (Fig. 2E,F) and the choroidal DPE (cDPE) where the choroid and scleral anterior margin became one spot (Fig. 2G,H).

Two different blinded authors (Y.P. and Y.J.A.) identified the rDPE, cDPE, disc center, fovea, and measured three parameters—distance, angle, and depth to quantify their 3D location relationship using the caliper function of the OCT program software. The schematic diagram of this relationship is shown in Fig. 3. Distances were measured between the disc and fovea, the DPE and disc, and the DPE and fovea. The disc-fovea angle was defined as the angle between the horizontal meridian across the OCT-defined disc center and the straight line from the center of the optic disc to the fovea. DPE-disc angle was defined as the angle between the DPE-disc and disc-fovea lines. The DPE-fovea angle was defined as the angle between the DPE-fovea and disc-fovea lines. Depth was calculated in micrometers (μm) after counting the separate image numbers for interface sections of each point and multiplying them by 2.6 times per count.

### Statistical analysis

Statistical analysis was performed using IBM SPSS for Windows version 22.0 (IBM Corp., Armonk, NY, USA). All continuous data were reported as the mean ± standard deviation (SD). Either a paired t-test or Wilcoxon signed-rank test was used to compare preoperative and postoperative values. An independent t-test or Mann–Whitney U test was used to compare the operated and non-operated eye, depending on the normal distribution measured by the Kolmogorov–Smirnov test. A p-value < 0.05 was considered statistically significant for all statistical analyses.

## References

[1] Bae SH, Choi DG (2018). Changes of corneal topographic measurements and higher-order aberrations after surgery for exotropia. PLoS ONE.

[2] Hong SW, Kang NY (2012). Astigmatic changes after horizontal rectus muscle surgery in intermittent exotropia. Korean J. Ophthalmol..

[3] Leshno A, Mezad-Koursh D, Ziv-Baran T, Stolovitch C (2017). A paired comparison study on refractive changes after strabismus surgery. J. AAPOS.

[4] Noh JH, Park KH, Lee JY, Jung MS, Kim SY (2013). Changes in refractive error and anterior segment parameters after isolated lateral rectus muscle recession. J. AAPOS.

[5] Rajavi Z, Mohammad Rabei H, Ramezani A, Heidari A, Daneshvar F (2008). Refractive effect of the horizontal rectus muscle recession. Int. Ophthalmol..

[6] Shin KH, Hyun SH, Kim IN, Paik HJ (2014). The impact of intermittent exotropia and surgery for intermittent exotropia on myopic progression among early school-aged children with myopia. Br. J. Ophthalmol..

[7] Hainsworth DP, Bierly JR, Schmeisser ET, Baker RS (1999). Corneal topographic changes after extraocular muscle surgery. J. AAPOS.

[8] Kwitko S, Feldon S, McDonnell PJ (1992). Corneal topographic changes following strabismus surgery in Grave's disease. Cornea.

[9] Preslan MW, Cioffi G, Min YI (1992). Refractive error changes following strabismus surgery. J. Pediatr. Ophthalmol. Strabismus.

[10] Krewson WE (1950). The action of the extraocular muscles: A method of vector-analysis with computations. Trans. Am. Ophthalmol. Soc..

[11] Rosen RB (2009). Multidimensional en-face OCT imaging of the retina. Opt. Express.

[12] Kim YC, Jung Y, Park HL, Park CK (2017). The location of the deepest point of the eyeball determines the optic disc configuration. Sci. Rep..

[13] Hsiang HW (2008). Clinical characteristics of posterior staphyloma in eyes with pathologic myopia. Am. J. Ophthalmol..

[14] Moriyama M (2011). Topographic analyses of shape of eyes with pathologic myopia by high-resolution three-dimensional magnetic resonance imaging. Ophthalmology.

[15] Clark RA, Demer JL (2006). Magnetic resonance imaging of the effects of horizontal rectus extraocular muscle surgery on pulley and globe positions and stability. Invest. Ophthalmol. Vis. Sci..

[16] Bixenman WW, von Noorden GK (1982). Apparent foveal displacement in normal subjects and in cyclotropia. Ophthalmology.

[17] Kothari MT, Venkatesan G, Shah JP, Kothari K, Nirmalan PK (2005). Can ocular torsion be measured using the slitlamp biomicroscope?. Indian J. Ophthalmol..

[18] Kushner BJ (1985). The role of ocular torsion on the etiology of A and V patterns. J. Pediatr. Ophthalmol. Strabismus.

[19] Emre S, Cankaya C, Demirel S, Doganay S (2008). Comparison of preoperative and postoperative anterior segment measurements with Pentacam in horizontal muscle surgery. Eur. J. Ophthalmol..

[20] Jung JH, Choi HY (2012). Comparison of preoperative and postoperative anterior segment measurements with Pentacam(R) in strabismus surgery. J. Pediatr. Ophthalmol. Strabismus.

[21] Chen K, Weiland JD (2014). Discovery of retinal elastin and its possible role in age-related macular degeneration. Ann. Biomed. Eng..

[22] Liu B, McNally S, Kilpatrick JI, Jarvis SP, O'Brien CJ (2018). Aging and ocular tissue stiffness in glaucoma. Surv. Ophthalmol..

[23] Ohno-Matsui K (2012). Association between shape of sclera and myopic retinochoroidal lesions in patients with pathologic myopia. Invest. Ophthalmol. Vis. Sci..

[24] Maldonado RS (2010). Optimizing hand-held spectral domain optical coherence tomography imaging for neonates, infants, and children. Invest. Ophthalmol. Vis. Sci..

